# Breast cancer biomarker detection through the photoluminescence of epitaxial monolayer MoS_2_ flakes

**DOI:** 10.1038/s41598-020-73029-9

**Published:** 2020-09-29

**Authors:** Sergio Catalán-Gómez, María Briones, Sandra Cortijo-Campos, Tania García-Mendiola, Alicia de Andrés, Sourav Garg, Patrick Kung, Encarnación Lorenzo, Jose Luis Pau, Andrés Redondo-Cubero

**Affiliations:** 1 Electronics and Semiconductors Group, Departamento de Física Aplicada, Universidad Autónoma de Madrid, 28049 Madrid, Spain; 2 Departamento de Química analítica y análisis, Universidad Autónoma de Madrid, 28049 Madrid, Spain; 3 Instituto de Ciencia de Materiales de Madrid, Consejo Superior de Investigaciones Científicas (ICMM-CSIC), C/Sor Juana Inés de la Cruz, 4, 28049 Madrid, Spain; 4 Electrical and Computer Engineering Department, University of Alabama, Tuscaloosa, AL USA

**Keywords:** Nanoscale materials, DNA and RNA, Breast cancer, Biomarkers, Materials science

## Abstract

In this work we report on the characterization and biological functionalization of 2D MoS_2_ flakes, epitaxially grown on sapphire, to develop an optical biosensor for the breast cancer biomarker miRNA21. The MoS_2_ flakes were modified with a thiolated DNA probe complementary to the target biomarker. Based on the photoluminescence of MoS_2_, the hybridization events were analyzed for the target (miRNA21c) and the control non-complementary sequence (miRNA21nc). A specific redshift was observed for the hybridization with miRNA21c, but not for the control, demonstrating the biomarker recognition via PL. The homogeneity of these MoS_2_ platforms was verified with microscopic maps. The detailed spectroscopic analysis of the spectra reveals changes in the trion to excitation ratio, being the redshift after the hybridization ascribed to both peaks. The results demonstrate the benefits of optical biosensors based on MoS_2_ monolayer for future commercial devices.

## Introduction

Breast cancer is the most frequent malignancy in women worldwide, with approximately one new case diagnosed every 18 s^1^. Reports show that the global incidence of breast cancer has been continuously rising, with annual increases of 3.1% since 1980, and more than 2 million women diagnosed every year. In fact, breast cancer contributes to about 23% of the cancer cases worldwide^2^. One of the essential steps to increase the survival rate in breast cancer is early diagnosis, since more than 90% of women diagnosed at the earliest stage survive their disease for at least 5 years, in comparison to around 15% for women diagnosed with the most advanced stage of the disease. Thus, the scientific community needs to put more emphasis on improving the diagnosis methods, especially through non-invasive procedures. In this context, a strong effort has been devoted to explore different routes for the detection of breast cancer using biosensors, which typically consists of a biomarker (target molecule), a bioreceptor (recognition element) and a compatible transducer (the platform used to convert biological into measurable signals)^3^.

Among most used biomarkers for breast cancer we can cite HER-2, BRCA1 and miRNA21^3^. miRNAs are an important class of small non-coding RNA involved in the regulation of gene expression, and whose mature products are around 18–25 nucleotides long. miRNA21 is one of the first human miRNA genes whose regulation has been extensively studied, because it has been found to be upregulated in many pathological conditions including cancer and cardiovascular^4^. It has been reported that miRNA21 is over expressed in many cancer tissues, such as gastric, lung, colorectal, prostate cancer^5^ but, more importantly, miRNA21 is the most targeted miRNA in breast cancer diagnosis because of its higher sensitivity (87.6%) and specificity (87.3%) at early stages^3^. In most of these cases, the typical bioreceptor is the complementary miRNA21 or a modified version of this, which is actually the approach followed in this work.

Concerning the transducers, in the last years the use of two dimensional (2D) materials has emerged as a promising route^6,7^. Among them, transition metal dichalcogenides, such as molybdenum disulfide (MoS_2_), have attracted intense attention as functional materials in a variety of biosensing applications^8^ and, particularly, for miRNA21 and DNA sensing^9–13^. Many of these strategies are focused on the electrochemistry, while others rely on optical transducers^14,15^. 2D MoS_2_ has a direct band gap in the visible range^16^ and a good chemical stability under ambient conditions^17,18^, what makes it a perfect candidate for photoluminescence (PL) biosensors. Moreover, after the interaction with the target sequence, the binding events occurring near the surface of MoS_2_ produce perturbations in the local dielectric permittivity, enabling detection via PL changes. However, the difficulty to produce high quality MoS_2_ flakes has limited the progress in this research line.

With the aim of taking advantage of MoS_2_ PL for the development of new biosensors for sensitive breast cancer biomarkers, in this work we use epitaxially grown MoS_2_ flakes on sapphire as transducers in a new biosensor for miRNA21 recognition. In order to functionalize these platforms with the bioreceptor (capture probe), we used a specific thiol modified DNA sequence. The thiol functional group is used to fix the biomolecule to the flakes^17–20^ in a disposition that allows hybridization with the target. For the assays, both complementary and non-complementary miRNA21 targets were employed, in order to demonstrate the selectivity of the biosensor. The characterization of the sensing response was carried out by individually monitoring the PL of MoS_2_ flakes. In addition, thanks to the microscopic PL system used, both the wavelength and the intensity were mapped across the surface of the flakes, analyzing the homogeneity of the response.

## Experimental section

### Materials

Concerning the transducer, the MoS_2_ powder of high purity (99%) used as the precursor for the growth of 2D flakes was obtained from Alfa Aesar. Sodium phosphate and sodium chloride were obtained from Scharlab Co. Synthetic 22-mer oligonucleotides were supplied by Sigma-Aldrich Co. A single-stranded DNA sequence modified at 5′-end with an hexalquilthiol was used as capture probe, and denoted as ss-DNAp-SH. As target analyte a fully complementary sequence (denoted as miRNA21c) and the non-complementary sequence (denoted as miRNA21nc) were used. All these sequences are listed in Table 1. All solutions were prepared just prior to use. Water was purified with a Millipore Milli-Q-system (18.2 MΩ·cm) and was sterilized with a Nüve OT 012 small steam autoclave.

**Table 1:** Synthetic oligonucleotides used in this work.

Nomenclature	Oligonucleotides sequence
ss-DNAp-SH	5′-SH(CH_2_)_6_–TCAACATCAGTCTGATAAGCTA
miRNA21c	5′-UAGCUUAUCAGACUGAUGUUGA
miRNA21nc	5′-AUCGAAUAGUCUGACUACAACU

### Procedures

#### Epitaxial growth of MoS_2_ monolayers

The MoS_2_ monolayers were synthesized by vapor phase growth on double side polished (0001) sapphire substrates, previously cleaned with acetone solution and isopropanol^21^. The growth was carried in a quartz tube fitted in a 3 zone furnace. MoS_2_ powder precursor was placed in a quartz boat at the center of the quartz tube. The sapphire substrates were placed downstream at a distance of ~ 10 cm from the precursor. The growth was performed at a pressure of 10 mbar under 20 sccm Ar flow, with the furnace temperature ramped to 970 °C and held there for a duration of 20 min. Afterwards, the furnace was cooled down naturally.

#### Immobilization of the thiolated capture probe onto MoS_2_ flakes

Prior to the capture probe immobilization, the prepared MoS_2_ flakes on sapphire were characterized by Raman and PL spectroscopy in order to confirm the monolayer growth and the PL intensity homogeneity. Then, 10 µL of a 10.0 nM thiolated capture probe (ss-DNAp-SH) solution was deposited onto the as-deposited MoS_2_ nanoflakes and was kept at 4 °C for 24 h. Afterwards, the functionalized MoS_2_ flakes (ss-DNAp-SH-MoS_2_) were washed with sterile water to remove unspecific adsorbed probe and was dried with N_2_ before PL experiments.

#### Hybridization event detection

The ss-DNAp-SH-MoS_2_ sensing platform was subsequently hybridized (1 h, 40 °C) with the analyte solution by addition of 10 µL of a 10.0 nM complementary (miRNA21c) or non-complementary (miRNA21nc) sequence in 10 mM phosphate buffer + 0.4 M NaCl pH 7.0 solution. The sensing platform was then immersed in sterile water to remove unspecific adsorbed material and dried with N_2_. Finally, the effect of the hybridization process in the MoS_2_ PL was studied.

#### Optical measurements

Raman and PL measurements were acquired in an Olympus (100 × objective) system with 488 nm laser at a power of 1 mW, a corresponding notch filter and a Jobin–Yvon iHR-320 monochromator (600 L/mm grating) coupled to a Peltier cooled Synapse CCD. With this system, PL maps were acquired with 1 µm steps in 10 × 10 µm^2^ areas. Each spectrum was taken at 1 s of integration time and 2 accumulations.

## Results and discussion

### Characterization of as-grown MoS_2_/sapphire platforms

**Figure 1:**
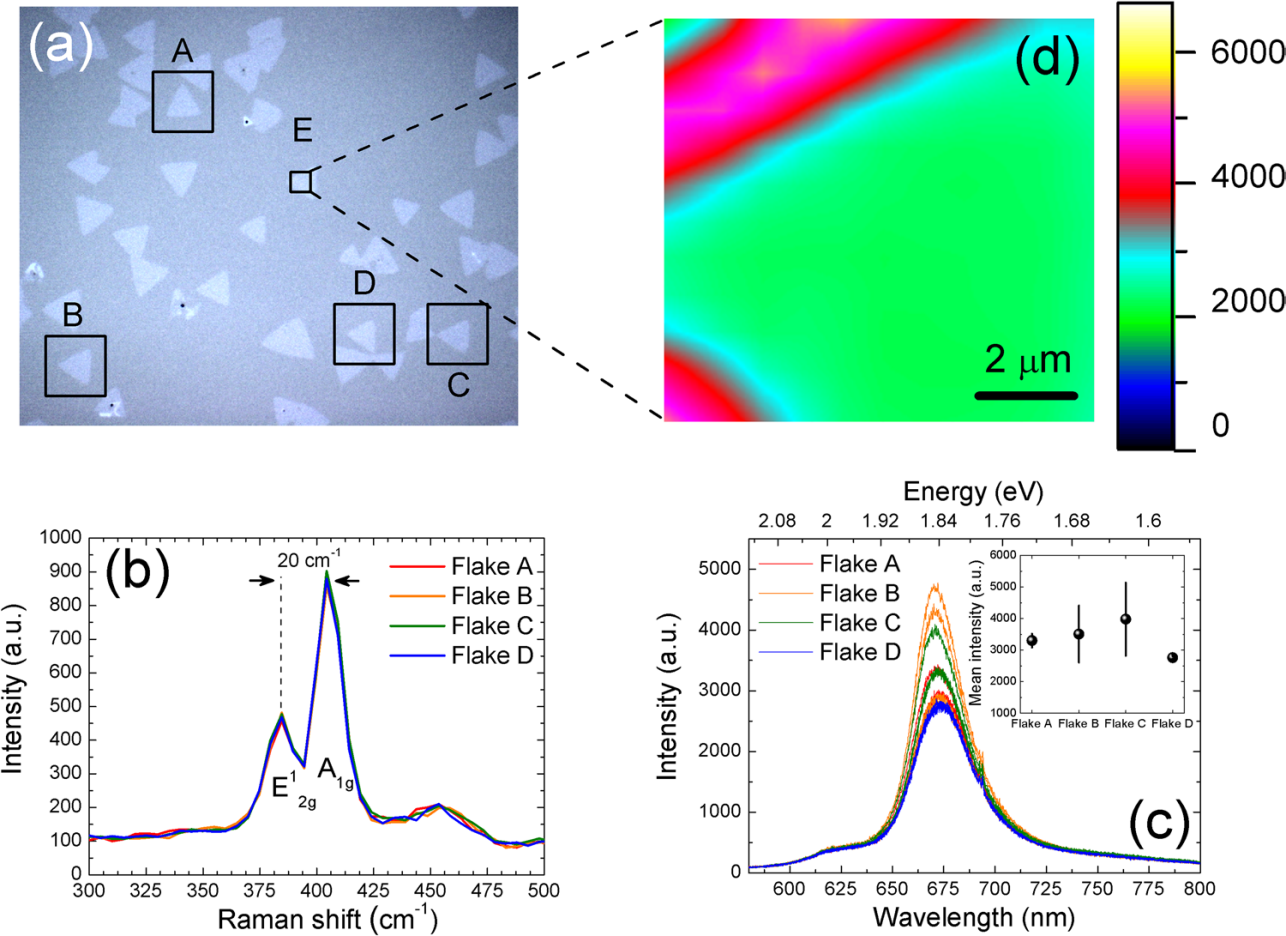
(**a**) Image of the MoS_2_ flakes grown on sapphire obtained by optical microscopy with a ×20 magnification. (**b**) Raman spectra of flakes A–D identified in panel (**a**), evidencing the same vibrational modes. (**c**) PL spectra of flakes A–D measured at the center. The inset shows the mean intensity taking into account the statistical error of the four spots measured. (**d**) Map of flake E from panel (**a**), showing the homogeneity of the PL signal at the center, and the increase at the border. Color scale indicates the intensity in arbitrary units.

In an optical biosensor, the quality of the platform is crucial for the subsequent understanding of the transduction procedure. In this work, the MoS_2_ material is epitaxially grown on sapphire by vapor phase transport. This method is commonly used for the deposition of large areas with good crystalline quality^22^. Figure 1a shows the optical image of one of these samples, displaying the well-known triangular morphology of MoS_2_ flakes, with dimensions of tens of microns. These flakes are composed of three layers of S-Mo-S in a sandwich-like structure named as monolayer MoS_2_. Different triangular isolated flakes (A–D) are identified in the figure. These flakes were analyzed by means of Raman and PL spectroscopy.

Figure 1b shows the Raman spectra of the different chosen flakes, which are almost identical and overlapped. The two main peaks are located at 384 cm^−1^ and 404 cm^−1^, which belong to the main phonon vibrations E^1^_2g_ and A_1g_, respectively^23^. The wavenumber difference between these two peaks (20 cm^−1^) is used as a fingerprint of the monolayer character of the material^24^, although some differences can arise from using different excitation lasers^23^ or substrates^25,26^. The ratio of the intensities A_1g_/E^1^_2g_ is 1.927.

The PL intensity is another indicator of the monolayer character^27^, and the results obtained in the flakes highlighted are shown in Fig. 1c. The spectra were acquired at four different points in the central regions of the flakes, and all of them display a broad band centered at ~ 673 nm, indicating the 2D character of the layers. The sharp peak around 693 nm in Fig. 1c belongs to impurities of Cr^3+^ from the sapphire substrate^28^. Here, the important fact is that the PL intensity barely changes between points (see the inset), confirming the similar quality of the flakes. However, in order to further analyze this homogeneity, we performed a detailed map of PL intensity in flake E (Fig. 1d). The intensity in the central part of the flake is essentially constant, and it increases considerably in the borders due to structural defects, typically Mo vacancies^21,29^.

### DNA functionalization and miRNA21 detection

**Figure 2:**
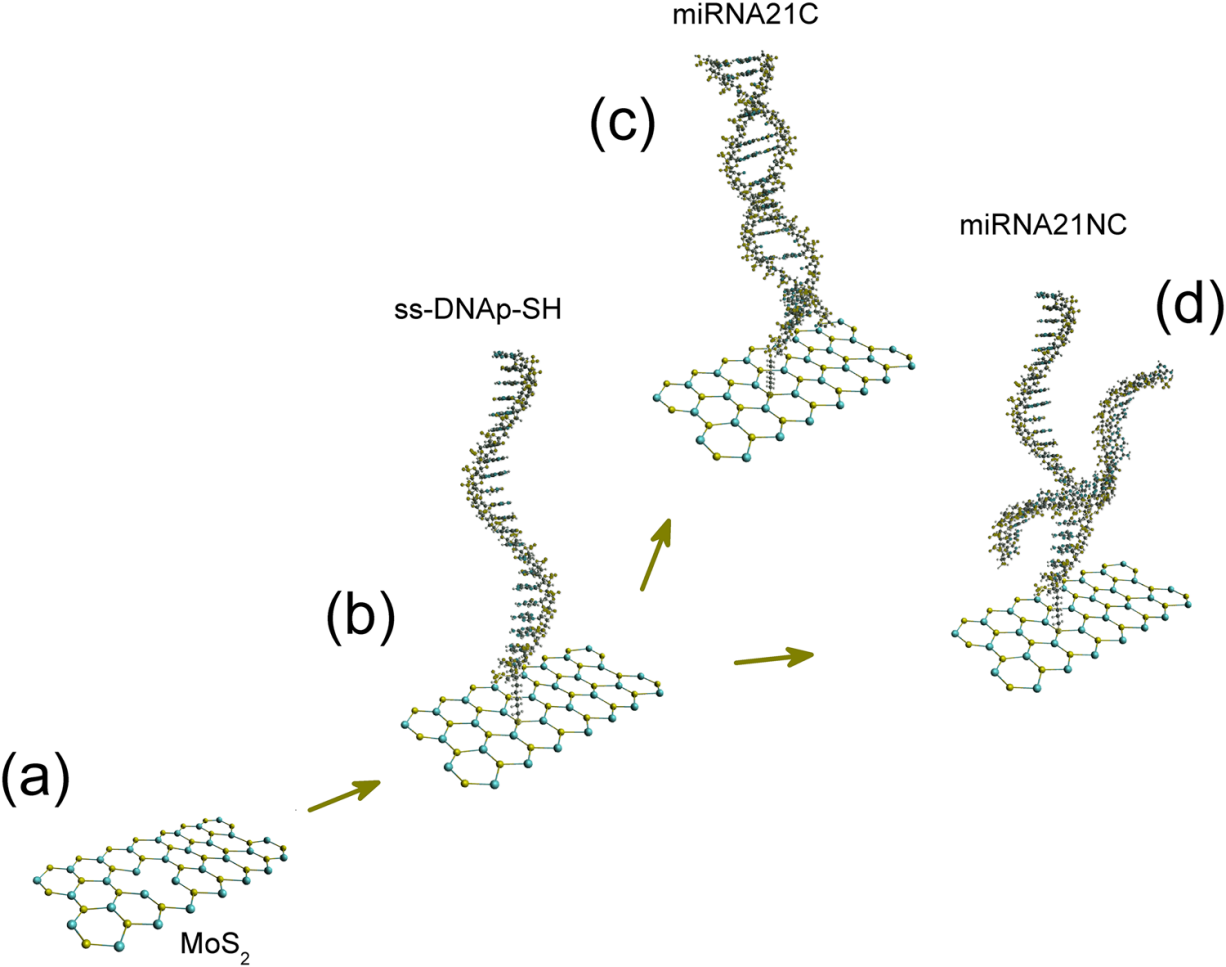
Scheme of the steps followed for the biosensing procedure. (**a**) Typical MoS_2_ surface, with the eventual presence of defects, (**b**) ss-DNAp-SH probe attached to the MoS_2_ surface. Hybridization on the MoS_2_ surface with (**c**) miRNA21c or (**d**) miRNA21nc.

The development of the MoS_2_ based biosensor is schematically shown in Fig. 2. Figure 2a shows the crystalline structure of the triangular MoS_2_ flakes deposited on sapphire. As can be seen in Fig. 2b, the first step is the functionalization of the triangular flakes deposited on sapphire with the DNA capture probe, a ss-DNA sequence totally complementary to the analyte (miRNA21c). Hence, the DNA probe modified at 5′-end with an hexalquilthiol (ss-DNAp-SH) is immobilized on MoS_2_ flakes through the thiol group. The organic molecules with -SH group tend to repair or eliminate S vacancies (V_S_) of the MoS_2_ lattice, resulting in the molecular functionalization with the substrate^19,20,30,31^. After this step, the functionalized platform was tested by hybridization with the complementary (miRNA21c) and the non-complementary (miRNA21nc) sequences, the latter used as control. Figure 2c,d shows these two hybridization assays, which were performed according to the procedure described in the experimental section. PL measurements have been taken in different MoS_2_ flakes before and after all these steps.

**Figure 3:**
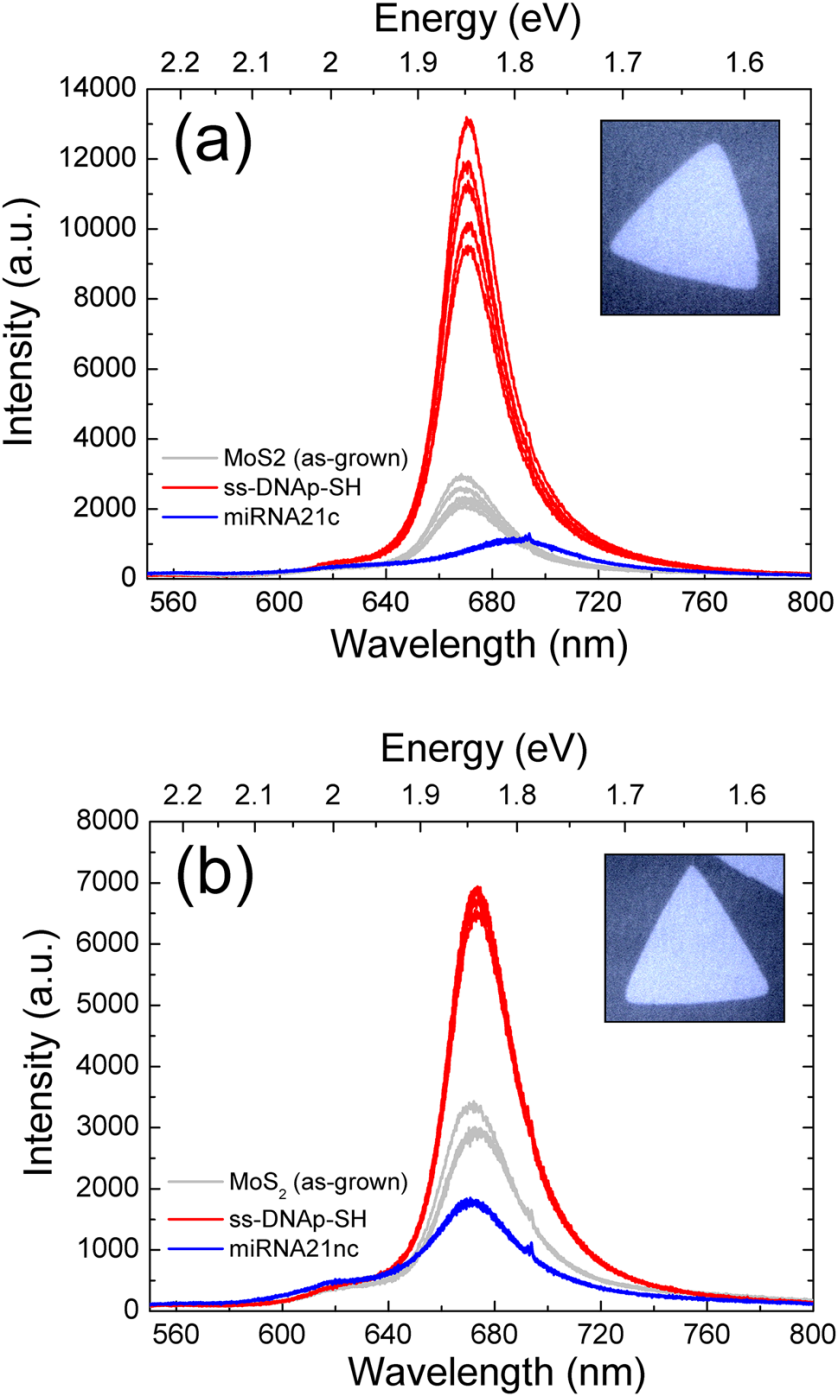
PL spectra for two flakes processed and tested with (**a**) miRNA21c and (**b**) miRNA21nc. Several spectra were acquired in different spots of the flake for the three steps (as-grown, ss-DNAp-SH functionalization, and target test). The insets are the optical images of the flakes used in the study (magnification ×100).

Figure 3 shows the PL spectra obtained for the two routes described before, carried out in two different flakes. As a standard procedure, we tagged different flakes for the analysis prior to the functionalization. Then, we acquired several spectra in different spots of the same flakes to analyze the reproducibility and increase the statistical significance.

Figure 3a shows the PL measurements for the as-grown MoS_2_ flake (grey lines), for the same flake after the immobilization of ss-DNAp-SH (red lines), and for the same flake after the hybridization with miRNA21c (blue line). Figure 3b shows the results for another flake where we used the miRNA21nc control.

A common feature is the increase of the PL after the immobilization of ss-DNAp-SH. Indeed, the intensity is enhanced by a factor ranging between 2 and 6. This fact indicates the effective anchorage of the probe to the MoS_2_ surface as discussed in detailed later on. However, the hybridization process is different for the miRNA21c (Fig. 3a) and for the miRNA21nc (Fig. 3b) targets. Although both of them evidence a decrease in the PL signal, the miRNA21c produces a singular redshift, which is not present in the miRNA21nc. It is important to mention that organic molecules can react with MoS_2_ by covalent chemical functionalization^32^ or by weak bonds such as Van der Waals^33^, resulting in non-specific reactions, but the redshift in the wavelength clearly points out that the bonding process is significantly different for miRNA21c than for miRNA21nc.

**Figure 4:**
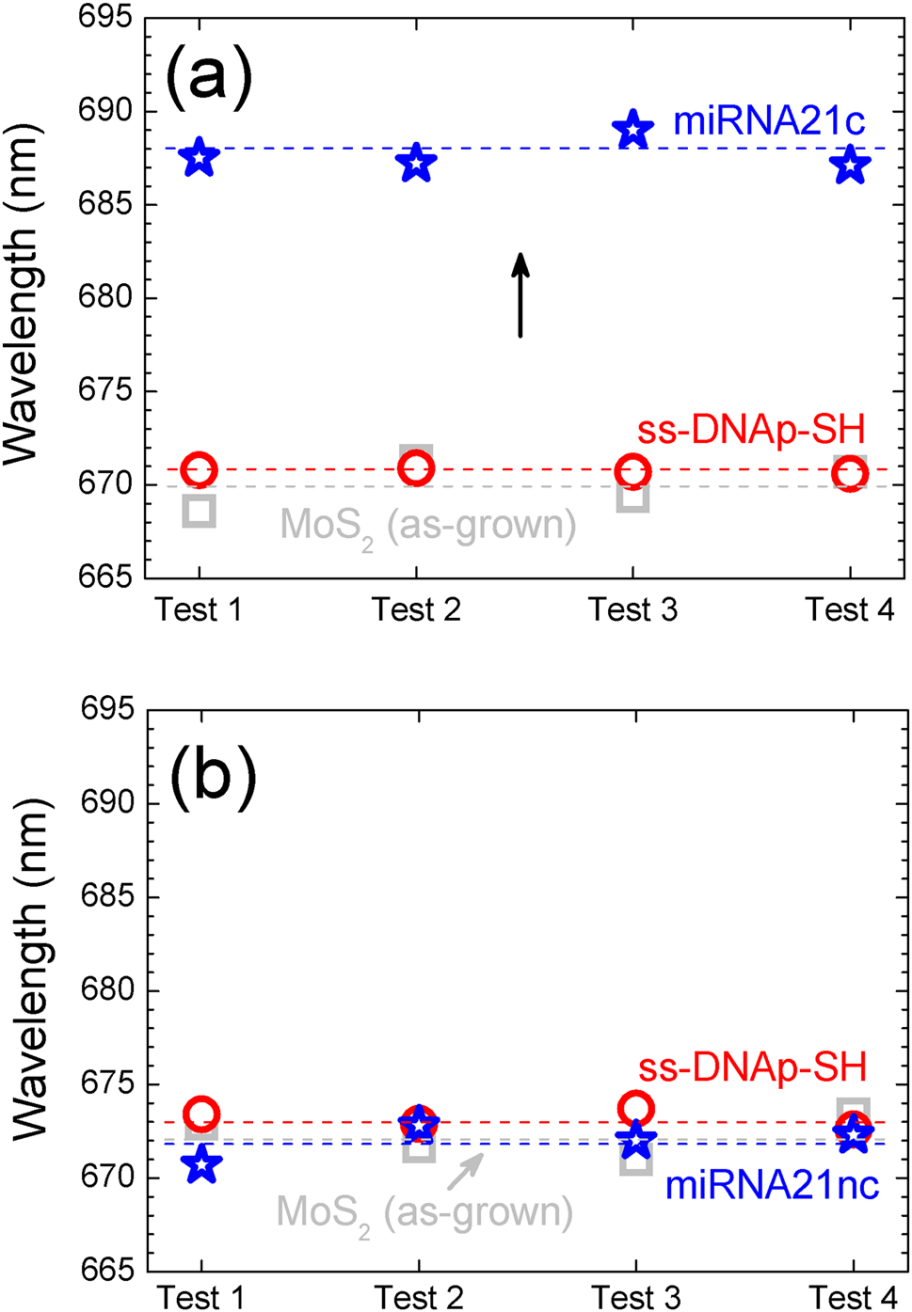
Results for the biosensing test in 4 different flakes for (**a**) miRNA21c and (**b**) miRNA21nc. Values are the average of the different spectra measured in each flake. A redshift of 16 nm takes place for miRNA21c while miRNA21nc shows no change. Statistical errors lie within the points of the graph.

In order to quantify this effect, Fig. 4 shows the wavelength of the PL peak for the different tests performed: miRNA21c (Fig. 4a) and the miRNA21nc (Fig. 4b). A redshift ~ 16 nm (43 meV) occurred for the miRNA21c sequence, whereas the miRNA21nc shows almost no changes. Note that we have performed these tests in 4 different flakes and 4 different spots for each flake, to warrant a good reproducibility of the data within the statistical errors. Therefore, the results demonstrate the specificity of the biosensor and the viability of the recognition of miRNA21 biomarker through PL.

**Figure 5:**
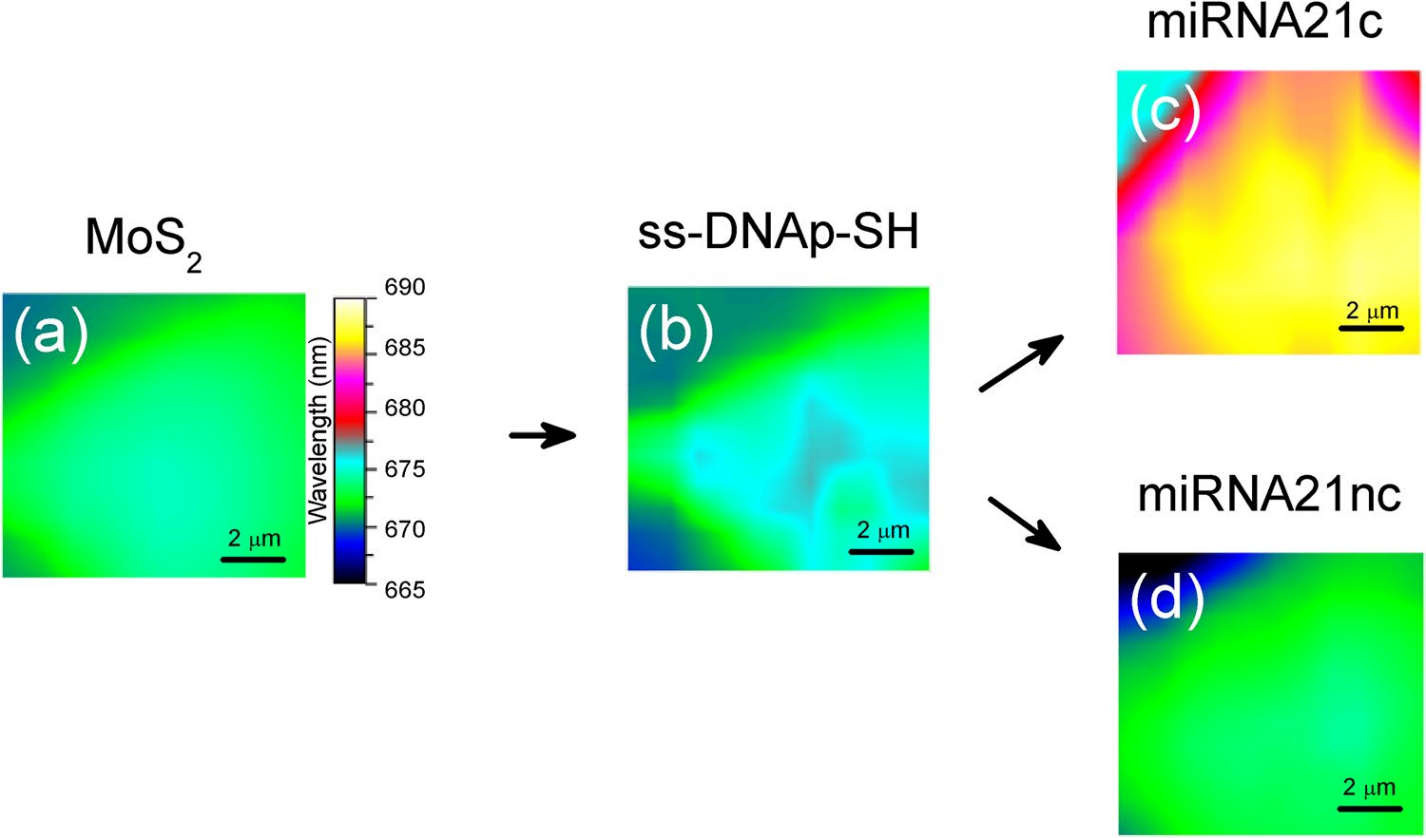
PL maps showing the wavelength variation in flakes (area of 10 × 10 μm^2^) for 4 different steps: (**a**) as-grown MoS_2_, (**b**) after ss-DNAp-SH functionalization, (**c**) after miRNA21c hybridization, (**d**) after miRNA21nc control test.

In order to further analyze the homogeneity of the biosensing platforms, we recorded PL wavelength maps for the different steps followed during the assays. Figure 5 shows the maps of the flakes used, where the color scale represents the wavelength of the main peak. Both as-grown MoS_2_ flakes (Fig. 5a) and ss-DNAp-SH functionalized flakes (Fig. 5b) show essentially the same PL emission, with maximum variations of 2 nm. When hybridization with the miRNA21c occurs (Fig. 5c), the PL map changes from cyan (670–675 nm) to yellow color (687–689 nm), indicating a homogeneous redshift on the surface of the flake. However, the hybridization with miRNA21nc (Fig. 5d) does not change the wavelength significantly.

It is important to note that the homogeneity is high in the central region, where most of the sulfur vacancies (V_s_) are found. At the borders of the MoS_2_ flakes the vacancies are predominantly Mo-based, as it has been demonstrated in different works^19,21,29^. Indeed, the borders exhibit p-type doping, typically ascribed to molybdenum vacancies (V_Mo_), which results in a blueshift of the PL main peak, an effect confirmed in our maps.

### Spectroscopic analysis of the PL peaks

As mentioned before, the functionalization of the MoS_2_ with the ss-DNAp-SH depends on the efficiency of the bonding to the as-grown MoS_2_ flakes. The presence of intrinsic defects of MoS_2_ monolayer has been intensively studied in the last few years, reporting a rich variety of point defects and dislocation cores^34,35^. These defects are known to have a relevant impact on the binding energies of the excitons^16^. Some of these defects have been identified in non-resonant PL studies at low temperatures^36^. Indeed, MoS_2_ monolayer seems to exhibit at least six optical transitions at 4 K, three of them ascribed to defects. However, at room temperature the remaining PL peaks are typically associated with three main optical states: the A^−^ trion (~ 1.85 eV), the A exciton (~ 1.90 eV), the B exciton (~ 2.03 eV)^36–39^. The negative trion is normally ascribed to the binding of the photoexcited electron–hole pairs with the excess electrons from the surface, i.e., to the presence of defects (normally producing the n-type behavior of MoS_2_)^40^. The exact energy values for the states, as well as the existence of multiexcitons are still under debate and may vary also for different substrates and experimental conditions. Multiexciton states, however, might be stable even at room temperature due to the strong Coulomb interaction and reduced dielectric screening of the monolayer flakes^37^.

**Figure 6:**
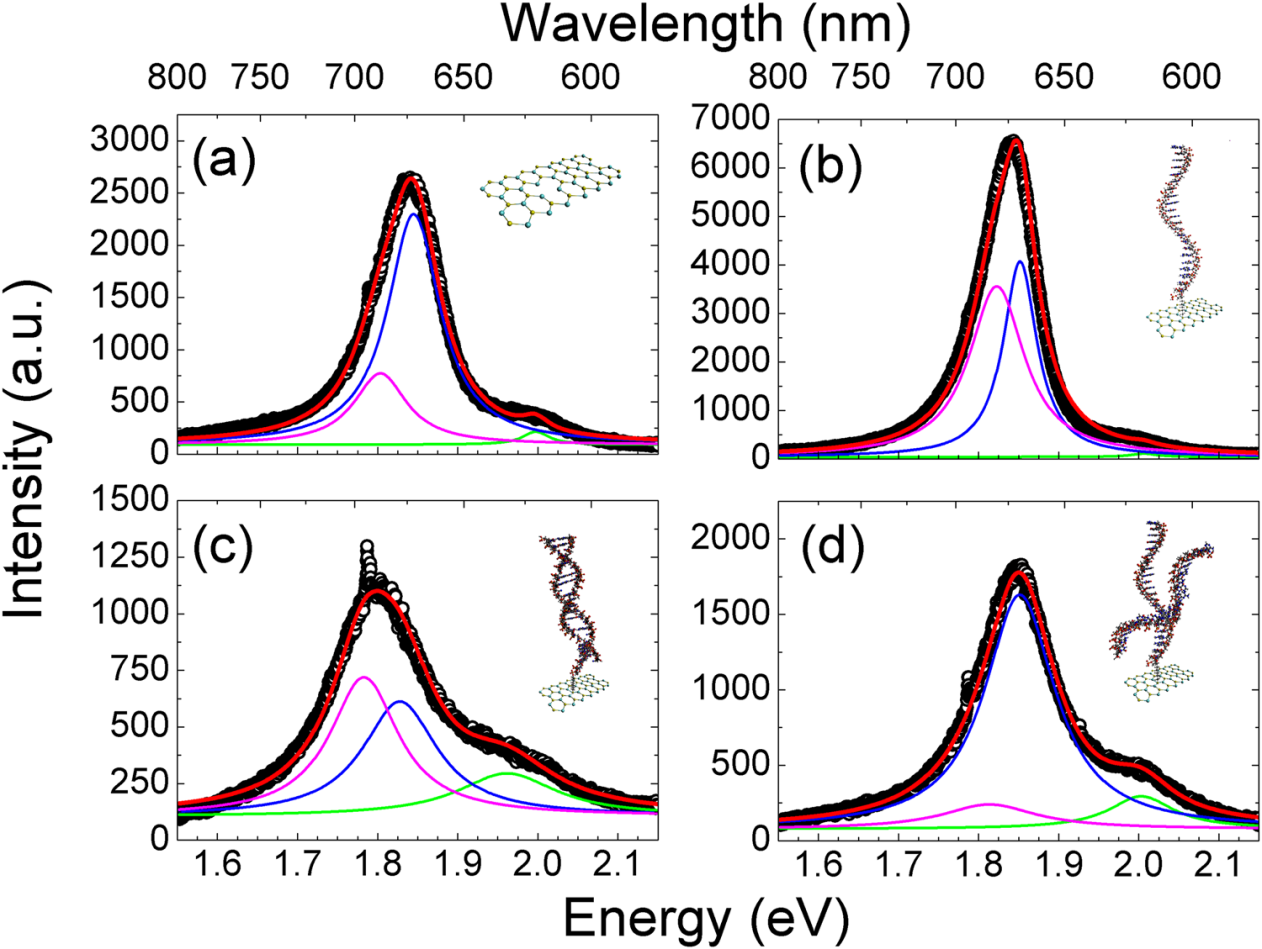
PL spectra for samples in a different stages: (**a**) pristine as-grown MoS_2_, (**b**) flake after ss-DNAp-SH functionalization, (**c**) after hybridization with the complementary sequence miRNA21c, and (**d**) with the non-complementary sequence miRNA21nc. The PL band is deconvoluted in the different contributions to fit the data.

Taking into account this scenario, we have carried out Lorentzian fits of representative PL spectra, shown in Fig. 6 and summarized in Table 2. Figure 6a shows the deconvolution of the PL peak for the as-grown MoS_2_ flake. Three main contributions have been identified at ~ 1.80, ~ 1.85, and ~ 2.00 eV, with the one at 1.85 eV the most intense. Thus, the most plausible assignment for the A exciton in our case is the peak at 1.85 eV and, correspondingly, the A^−^ trion at 1.80 eV and the B exciton at 2.00 eV. The values for the A exciton and A^−^ trion agrees well with the ones reported in similar MoS_2_ flakes by Zuo et al.^41^.

**Table 2:** Peak positions corresponding to the PL analysis in Fig. 6.

Sample	A^−^ trion (eV)	A exciton (eV)	B exciton (eV)
As-grown MoS_2_	1.80(4)	1.85(1)	2.00(2)
With probe ss-DNAp-SH	1.82(4)	1.85(3)	2.01(2)
Complementary (miRNA21c)	1.78(5)	1.83(6)	1.96(8)
Non-complementary (miRNA21nc)	1.81(7)	1.85(5)	2.00(5)

Figure 6b shows the analysis of a characteristic PL spectrum after the ss-DNAp-SH functionalization. As mentioned in the previous section, the intensity of the PL emission is higher. An increase of the MoS_2_ PL emission intensity is often attributed to the chemical adsorption of molecules, although this effect can depend on the particular molecule. The experiments performed with single-stranded and double-stranded DNA proved the good affinity of such biomolecules to MoS_2_ via van der Waals forces^11^. More importantly for our case, the thiol groups may repair the S vacancies or transfer electrons to MoS_2_, causing the passivation of defect mediated non-radiative recombination or provide excess electrons in the conduction band of MoS_2_^30,42^. Therefore, the PL enhancement we see can be understood as a result of the effective functionalization of the bioreceptor through both specific (thiol) and weak bonding.

From Fig. 6b, we see that the optical mechanism behind the PL change has two clear features: the overall improvement of the intensity and the change in the A^−^/A ratio. These effects are compatible with screening mechanisms due to the change of the dielectric function produced by the functionalization^39^. At the same time, charge transfer mechanisms between MoS_2_ monolayer and other molecules (or layers) have been reported recently^43,44^ and validated by means of density functional theory computations^45^. Interestingly, this charge transfer mechanism was found to be substrate-dependent too^26^. Therefore, a combination of these two phenomena (change in the dielectric function and charge transfer) can explain the results found.

Figure 6c shows the PL of the hybridized platform with miRNA21c, evidencing an overall decrease of the intensity and a redshift. The decrease in the intensity can be interpreted as the presence of non-radiative channels. The redshift involves both the displacement of A^−^ and A peaks but, also, a new change in the A^−^/A ratio, which is higher than unity. In addition, the B exciton also experiences a redshift. This behavior does not occur with the miRNA21nc control (Fig. 6d), where the trion peak intensity decreases significantly in the spectrum. Due to the broad features of the spectra, the existence of an additional peak cannot be completely ruled out (a good fit is also possible with 4 peaks in Fig. 6c,d). Hence, the reasons for the observed behavior are not clear and, despite the recent progress^36,37,46^, the current reports does not allow determining a unique origin for that. More research is needed to establish the exact energies of the excitons and multiexcitons, and the specific effect of defects on the optical properties. In this sense, the use of combined and more local techniques could help to shed light on these issues.

## Conclusions

In this work we have fabricated an optical biosensor for miRNA21 biomarker of breast cancer taking advantage of the PL of MoS_2_ monolayer flakes. This sensor has been produced in three steps: (1) growth of MoS_2_ epitaxial layers on sapphire, (2) functionalization with a thiolated DNA probe (ss-DNAp-SH), and (3) hybridization with a complementary and non-complementary miRNA21 sequences (the latter used as a control). The modification with the ss-DNAp-SH increases the native PL from MoS_2_, which diminishes after the recognition assays. A redshift of 16 nm was observed exclusively for the hybridization with miRNA21c, but not for the control miRNA21nc sequence, demonstrating the specificity of the biosensor and the viability of the recognition via PL. The homogeneity of the biosensing platforms was further verified with microscopic maps. The detailed spectroscopic analysis of the spectra reveals changes in the A^−^/A trion/exciton ratio, with the redshift after the hybridization ascribed to both peaks. Overall, our results indicate the benefits in terms of sensitivity and selectivity of optical bionsensors based on MoS_2_ monolayer. The transduction method through the PL wavelength change, instead of the PL intensity, is a significant achievement for the development of commercial biosensors in the future. Due to the use of individual flakes for the tests only a small area of the sample is needed. However, more research is needed to recover the sensor to the original stage after its usage, since the functionalization of the probe and the miRNA21 modify the original PL intensity of the flakes, preventing the recycling of the same flakes.
